# Insomnia in older HIV-positive patients

**DOI:** 10.5935/1984-0063.20220064

**Published:** 2022

**Authors:** Cristina Salles, Miguel Meira e Cruz, Igor Cardoso Freire, Lucas Haine Gonzaga, Cristiane Costa Dias

**Affiliations:** 1 Bahiana School of Medicine and Public Health, Salvador - Bahia - Brazil; 2 Centro Cardiovascular da Universidade de Lisboa, Lisbon School of Medicine, Sleep Unit - Lisbon - Portugal

**Keywords:** HIV, Sleep Disorders, Aged

## Abstract

**Introduction:**

Insomnia is a common sleep disorder in elderly. Although the HIV-positive population have a similar life expectancy when compared to the general population, some factors may interact with immunity conditions and therefore contribute to a worse prognosis.

**Objective:**

This is a review of literature that aims to identify prevalence of insomnia in older HIV-positive patients.

**Material and Methods:**

This is a review of literature conducted by using MEDLINE-PubMed, Embase, Cochrane Library, CINAHL, Web of Science, Scopus, SciELO, LILACS, and VHL databases, in addition to conducting manual searches. The terms used for the search were related to prevalence, HIV, insomnia, and advanced age. Inclusion criteria were: cross-sectional, cohort, and longitudinal studies, patients with a previous diagnosis of HIV in old age, studies reporting the frequency of insomnia or insomnia symptoms. The criteria for exclusion were: clinical trials, animal studies, letters, abstracts, conference proceedings, studies with other sleep scales that did not include insomnia.

**Results:**

There were 2,805 publications found in the database and a further 10 articles were included manually. Of this total, four were included in this review, resulting in a total of 2,227 participants. The prevalence of insomnia in HIV-positive patients over 50 years varied from 12.5% to 76.5%.

**Conclusion:**

The frequency of insomnia was higher in the profile of the population studied than in the general population. This should be clinically relevant in order to adequately treat and impact on the prognosis of those patient.

## INTRODUCTION

The human immunodeficiency virus (HIV) is responsible for causing the acquired immunodeficiency syndrome (AIDS), identified in the late 70’s and early 80’s^1^. According to UNAIDS data, in 2018, 37.9 million people lived with HIV (PLWH) in the world^2^. Currently, HIV is still a disease with no cure (although there are treatments for a lifetime) and surrounded by taboos. These characteristics can be considered propelling factors of emotional stress, making HIV a disease that brings suffering to patients and leading to the hypothesis that these facts predispose to insomnia in HIV-positive patients^3^.

The early and adequate treatment of PLWH results in better outcomes, particularly regarding with life expectancy of these patients, which may turn similar to life expectancy of general population^4^. Insomnia is a disorder that becomes more frequent with age^5^; together with multiple drug use, fatigue related complaints, comorbid psychiatric disorders, insomnia may be frequent in HIV-positive older patients.

On the other hand, inadequate sleep including insomnia, is linked to dysfunctional immunity and disturbed immunity response in immunosuppressed patients^6^. As this interaction may have clinical significant impact in the context of HIV infection, it is relevant to understand the prevalence. The aim of this study was review the prevalence of insomnia in older HIV-positive patients.

## MATERIAL AND METHODS

Searches on MEDLINE/PubMed, Embase, The Cochrane Library, CINAHL, Web of Science, Scopus and Virtual Health Library (VHL) electronic data sources were performed through a combination of descriptors, including terms from Medical Subject Headings (MeSH), Health Science Descriptors (DECs) and descriptor contractions. The review of literature was not restricted to English publications since studies written in Portuguese and Spanish were also included. The PRISMA protocol was used as a guide for the review of literature.

The terms used for the search were related to prevalence, HIV, insomnia, and advanced age. The combination of descriptors resulted in: (“epidemiology”[Subheading] OR “epidemiology”[All Fields] OR “prevalence”[All Fields] OR “prevalence”[MeSH Terms]) AND (“sleep initiation and maintenance disorders”[MeSH Terms] OR (“sleep”[All Fields] AND “initiation”[All Fields] AND “maintenance”[All Fields] AND “disorders”[All Fields]) OR “sleep initiation and maintenance disorders”[All Fields] OR “insomnia”[All Fields]) AND (“hiv”[MeSH Terms] OR “hiv”[All Fields]) AND (“aged”[MeSH Terms] OR “aged”[All Fields] OR (“advanced”[All Fields] AND “age”[All Fields]) OR “advanced age”[All Fields]).

### Inclusion and exclusion criteria

The inclusion criteria were: cross-sectional, cohort, and longitudinal studies. The accepted data were in patients with the previous diagnosis of HIV in advanced age, those over 50 years; studies that report the frequency of insomnia or insomnia symptoms (accepted symptoms: difficulty in starting sleep, difficulty in maintaining sleep, multiple awakenings during sleep and early awakening).

The criteria for exclusion were: clinical trials, animal studies, letters, abstracts, conference proceedings, studies with other sleep scales that did not include insomnia.

Identification and selection of studies were made separately, and the pre-selected titles and abstracts were read to identify only those studies that correctly met the inclusion criteria. The reading of the articles continued in order to ensure the criteria for the review of literature.

The STROBE tool evaluated the quality of each study to assess the risk of bias of observational studies. Studies contemplating at least 70% of the questions in the STROBE tool were accepted.

## RESULTS

**Figure 1:**
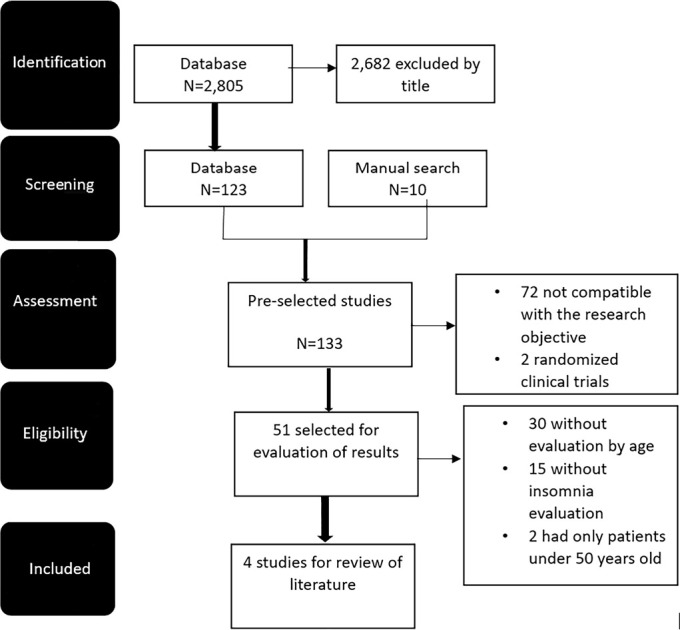
Flowchart of the study selection process.

A total of 2,805 publications were identified in the database. After reading the titles, 123 were selected to read the abstract and added another 10 articles obtained through manual searches. Of these, 51 were pre-selected for data and results evaluation. The reading of the results allowed 4 studies to be retained for evaluation and 47 others to be excluded. Of the excluded studies, 30 did not address insomnia by age, therefore not allowing the cutting of insomnia in advanced age patients. Fifteen other studies did not address insomnia, and two other studies had patients only under 50 years old. Figure 1 shows the flowchart.

Table 1 details the included studies assessment using the STROBE tool, with articles covering at least 70% of this tool’s requirements being accepted.

**Table 1:** Assessment of the selected studies quality based on the STROBE tool.

Topic	STROBE item	Elliot et al.^7^	Jean-Louis et al.^8^	Ding et al.^9^	Saberi et al.^10^
**Title and abstract**	1	●	✓	●	✓
**Introduction**					
Background/rationale	2	✓	✓	✓	✓
Objectives	3	✓	✓	✓	✓
**Methods**					
Study design	4	✓	✓	✓	✓
Setting	5	○	✓	✓	✓
Participants	6	✓	●	✓	✓
Variables	7	○	○	✓	●
Data sources/measurement	8	✓	✓	✓	○
Bias	9	●	✓	●	●
Study size	10	✓	●	✓	●
Quantitative variables	11	✓	✓	✓	✓
Statistical methods	12	✓	✓	✓	●
**Results**					
Participants	13	✓	✓	✓	✓
Descriptive data	14	✓	✓	✓	✓
Outcome data	15	✓	✓	✓	●
Main results	16	✓	✓	✓	✓
Other analyses	17	○	○	✓	✓
**Discussion**					
Key results	18	✓	✓	✓	✓
Limitations	19	✓	✓	✓	✓
Interpretation	20	✓	●	✓	✓
Generalizability	21	○	●	✓	✓
**Other information**					
Funding	22	✓	✓	✓	✓
**Score**		90,9%	81,8%	90,9%	77,3%

Table 2 summarizes the studies.

**Table 2:** General characteristics of the studies ordered by year of publication.

Authors	Country, year	Type of study	N	N, HIV + over 50 years old	Evaluation of insomnia
Jean-Louis et al.^8^	EUA, 2012	Cross-sectional study	1682	Not detailed	Insomnia symptoms
Saberi et al.^10^	EUA, 2013	Cross-sectional study	14	4	Insomnia symptoms
Ding et al.^9^	China, 2018	Cross-sectional study	488	87	Insomnia symptoms
Elliot et al.^7^	EUA, 2019	Longitudinal study	43	43	ISI scale

Notes: ISI = Insomnia severity index; HIV = Human immunodeficiency virus.

**Figure 2:**
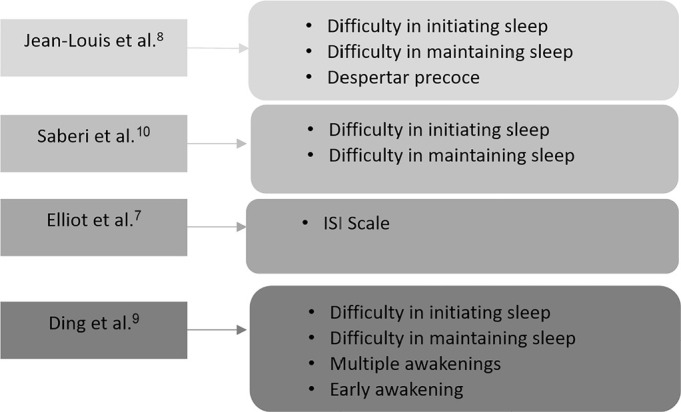
The main ways to approach insomnia or insomnia symptoms.

The main ways to approach insomnia or insomnia symptoms, based on questions or the insomnia severity index (ISI) scale, are summarized in Figure 2 separately by the author’s name.

**Figure 3:**
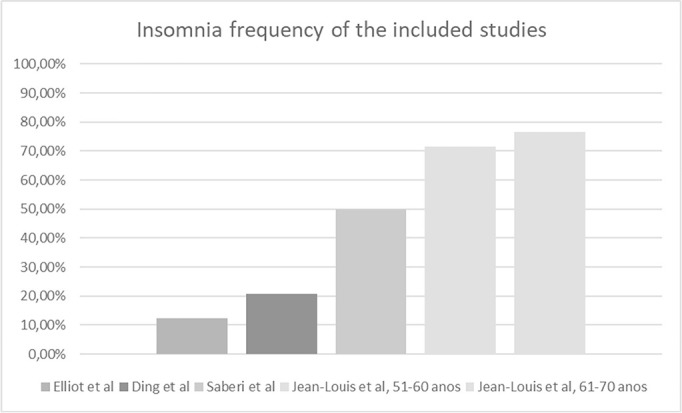
Insomnia frequency of the studies included in the review of literature.

Figure 3 shows the prevalence of insomnia in the elderly HIV-positive population in each included study. The research by Jean-Louis et al. (2012)8 is demonstrated according to the age groups.

### Included studies

Elliot et al. (2019)^7^ conducted a 180-day longitudinal study with 43 patients previously diagnosed with HIV and over 60 years old, the median being 66 years. Of these, 40 completed the minimal assessment of the study, i.e., the first 28 days according to the criteria. The research objective was to evaluate the serum concentration of dolutegravir, an antiretroviral drug of the integrase inhibitors class, in these patients on pre-selected days. The study included participants willing to take part in it. A total of 5 patients reported moderate or severe insomnia during the study, representing an insomnia frequency of 12.5% in this specific sample. The criteria for insomnia in this study was the ISI scale.

Ding et al. (2018)^9^ conducted a cross-sectional study between 2014 and 2015 with all patients over 40 years of Taizhou prefecture (China) previously diagnosed HIV-positive. A total of 488 participants were analyzed, 244 of whom were HIV-positive and the remainder HIV-negative. The study’s main objective was to analyze the association between insomnia and the short size of telomeres in HIV-positive patients. Among the patients diagnosed with HIV, 87 were over 55 years old, meeting the evaluation criteria of this review of literature, and among these, 18 patients had insomnia symptoms, representing a frequency of 20.7%. In order to evaluate the criteria of insomnia symptoms, four questions were asked to the participants regarding their sleep in the last month: “Did you have difficulty falling asleep? Did you wake up too early and were unable to go back to sleep? Did you have difficulty in staying asleep? Did you wake up several times during your sleep?” There were 4 possible answers, common to all questions: no, some nights, most nights, every night. It was defined as symptomatic for insomnia those patients who answered “most nights” or “every night” for at least two questions.

Jean-Louis et al. (2012)^8^ conducted a cross-sectional study of 1,682 HIV-infected and non-HIV-infected women, 69% of whom were previously diagnosed with HIV. The principal analysis of the data was to evaluate age-related insomnia symptoms in the selected group. The presence of one or more of the three criteria was defined as an insomnia symptom: difficulty in initiating sleep, difficulty in maintaining sleep, or awakening early three times or more during the week in the two weeks before the interview. This study gathered the participants into age groups and did not reveal the number of each of these groups and only reported the prevalence of insomnia symptoms on a chart without mentioning the exact number of symptomatic women. The range of 51 to 60-year-old women had approximately 71.4% insomnia symptoms, while 61 to 70-year-old women had approximately 76.5% insomnia symptoms.

Saberi et al. (2013)^10^ conducted a qualitative study with 14 HIV-positive participants, four of whom were over 50 years old, and two of whom had insomnia symptoms, resulting in a 50% prevalence. Insomnia was considered in these patients because both reported difficulty initiating and maintaining sleep. The evaluation of the participants was done through qualitative interviews. The investigation’s main objective was to evaluate the relationship between sleep quality and poor therapeutic compliance with HIV treatment in an individualized manner.

From the studies presenting patients also HIV negative for control and comparison (Ding et al. (2018)^9^ and Jean-Louis et al. (2012)^8^), there was no statistical significance of the difference between the presence of insomnia in the HIV-positive and HIV-negative groups in the age groups that adapted to this review of literature.

Jean-Louis et al. (2012)^8^ reported that the major limitation of the study was the absence of an approach to daytime dysfunction related to insomnia. There might also be a selection bias since only women who were already part of a cohort participated in the investigation.

Ding et al. (2018)^9^ also reported the issue of not addressing daytime dysfunctions for the evaluation of insomnia. Furthermore, they mentioned the problematic approach of separating insomnia from depression since they are very often comorbid.

Elliot et al. (2019)^7^ concluded that their study’s major problem was the recall bias mainly due to questionnaires were self-answered by the survey participants.

Saberi et al. (2013)^10^ described that the study bias was that all participants already had a diagnosis of poor sleep quality and mentioned the relationship between sleep disorders in general and depression, similar to Elliot et al. (2019)^7.^

## DISCUSSION

In this review of literature, it was possible to observe that the prevalence of insomnia and insomnia symptoms ranged from 12.5% to 76.5% in HIV-positive patients over 50 years old. Among the research included, the study by Jean-Louis et al. (2012)^8^ presented the highest prevalence with 76.5%; however, we would like to consider that the sample comprising this study was women, which may account for this higher prevalence. Followed by Saberi et al. (2013)^10^ with 50%, in third place was the investigation by Ding et al. (2018)^9^ with 20.7%, and finally, the lowest prevalence was the study by Elliot et al. (2019)^7^ with 12.5%. It is estimated that 10 to 30% of the general population has some insomnia symptoms^11^, compared to the studies of this review of literature, which all were above 10%. Results revealed a higher insomnia frequency in the studied population than it was expected from the general population, showing the relevance of this topic in this particular group of patients. Therefore, sleep evaluation and early detection of sleep disorder in this group of individuals becomes mandatory in order to achieve optimal therapeutical results. The high prevalence of insomnia in HIV-positive elderly individuals is also important due to their interacting role with immunity, systemic inflammation and coagulation, which are critical risk factors in this particular age group^6^,^12^,^13^.

In general, sleep has a restorative function concerning the immune system; therefore, reducing total sleep time could lead to immunological deficiencies. Studies have shown that reduced (<6h) or excessive (>9h) sleep time is associated with an increased risk of cardiovascular events and mortality^14^. Irwin et al. (1996)^15^ showed that prolonged and severe sleep deprivation is associated with alterations in natural and cellular immune function. These authors evaluated whether alterations in immune function also occur after modest sleep deprivation and also assessed the effects of partial sleep deprivation in the early evening on circulating leukocyte numbers, natural killer (NK) cell numbers, and cytotoxicity, cellular activity, and interleukin-2 (IL-2) production. For this purpose, they conducted a study on 42 healthy male volunteers who were submitted to one night of sleep deprivation between 10 p.m. and 3 a.m., and a reduction in natural immune responses was observed. In addition, IL-2 production was suppressed after sleep deprivation. After recovering from the night’s sleep, NK activity returned to basal levels and IL-2 production remained suppressed. These data reinforce the importance of sleep in modulating immunity and demonstrate that even modest sleep deprivation can reduce natural immune responses and T-cell cytokine production. These results are in line with the studies by Hall et al. (2015)^16^, who followed up 3,000 elderly people for nine years and observed an association between markers interleukin-6 (IL-6), tumor necrosis factor-alpha (TNF-α), and C-reactive protein (CRP) with reduced hours of sleep and increased mortality. Regarding measures of cellular immunity, the population with reduced sleep hours (<8h) showed progressive increases in the number of leukocytes, neutrophils, and monocytes^17^. Smagula et al. (2016)^18^ studied 2,500 elderly people for more than seven years and observed that when the elderly presented reduced total sleep time (<5h), there was an association with increased pro-inflammatory substances such as CRP, IL-6, and TNF-α, as well as increased mortality in this subgroup^18^,^19^. Thus, it can be seen that sleep disorders can significantly impact immune system function since sleep deprivation is directly associated with an increase in pro-inflammatory cytokines, such as IL-6 and TNF-α, and a decrease in circulating killer cells. This relationship between sleep and immunity has already been studied regarding telomere length in immunocompetent cells, which is considered a sign of aging immunity. Studies reported by Jackowska et al. (2012)^20^, including men who slept less than 5h, found that telomere length reduction was 6% compared to those who slept 7h or more.

Female is considered a predisposing factor for insomnia. In this review of literature, we could be observe that the study by Jean-Louis et al. (2012)^8^ had the highest frequency of insomnia symptoms and was carried out only with the female population. This study obtained frequencies higher than 70% of insomnia symptoms in women aged 50 to 70 years. The prevalence of insomnia symptoms in the general female population may reach 57% in women aged 18 to 60 years^21^; however, when studying the prevalence of insomnia symptoms in women over 65 years, the prevalence may exceed 70%^22^. Jean-Louis et al. (2012)^8^ and Jaussent et al. (2011)^22^ indicate that insomnia is prevalent in older women regardless of HIV status.

This differentiation in the prevalence of insomnia between the genders is essential because the inflammatory system differs between men and women^23^. Women tend to have more pronounced inflammatory responses than men, although this tendency is reversed both in childhood and in the elderly. There seems to be a hormonal relationship: estrogen would have a dose-dependent effect on the inflammatory system, such that low doses would lead to increased levels of IL-6, IL-1β, and TNF-α, while high levels could suppress this inflammatory activity^23^. Although it seems that sleep disorders are associated with higher levels of circulating IL-6 in women than in men, it is not yet well defined^24^.

The prevalence of insomnia was studied separately in the general population by Ohayon (2002)^25^, and he concluded that the prevalence could vary. When only insomnia symptoms were analyzed, the presence in the population was from 30 to 48%. When associated the insomnia symptom with a minimum frequency of three times a week, this prevalence decreased to the range of 16-21%; when questioned the presence of insomnia symptoms and daily dysfunction, it varied between 9-15%; whereas, the insomnia diagnosis was 6% in the general population. In Ohayon’s investigation, the criteria for insomnia diagnosis were the same as for DSM-IV and DSM-V. This factor is explicit in this review of literature since the studies that addressed only insomnia symptoms showed the highest frequencies: Saberi et al. (2013)^10^ and Jean-Louis et al. (2012)^8^. The study by Ding et al. (2018)^9^ also evaluated insomnia symptoms associated with other symptoms. These characterized the participants with insomnia only when they answered that they had two of the four symptoms investigated in most nights of sleep or every night, in 50% of the approached questions. The lowest frequency was in the study by Elliot et al. (2019)^7^, who used the ISI scale for diagnosis, compatible with Ohayon’s evaluation. In order to clarify the prevalence and frequency more precisely of insomnia, it is necessary to use standardized clinical criteria.

An important limitation among the studies, which were excluded, was that they did not address insomnia or sleep disorders specifically, but the sleep quality more widely. Several articles were excluded from this review of literature because they used only the total value of the Pittsburgh sleep quality index (PSQI) or other scores. This score has appropriate practical usefulness in general; however, the evaluation is to characterize good sleepers and poor sleepers. Mollayeva et al. (2016)^26^ consider that the PSQI is challenging to compare populations at the expense of the tool’s scope. Further research after stratification on the PSQI scale is needed for more accurate diagnosis and management.

The association between insomnia and depression is often comorbid. Among the studies that address concomitant depression with insomnia, the study by Saberi et al. (2013)^10^ comprises all 14 patients of the study with depression or depressive symptoms, showing a considerably higher frequency when compared to the other studies of this review of literature, as Jean-Louis et al. (2012)^8^ with a frequency of 27.6% presenting this association and Ding et al. (2018)^9^ with 14.75%. The association between depression and insomnia is frequent. In a study with 994 participants with a previous diagnosis of depression, 93% of the patients presented insomnia symptoms^27^. Another meta-analysis identified a 39.1% prevalence of depression in HIV-positive patients^28^. Data from these different studies show high frequency of depression and insomnia in HIV patients. An elderly HIV-infected patient may be potentially predisposed or in conditions likely to precipitate sleep disturbances due to psychosocial stress or anxiety from the moment they are facing a highly complex disease, or may precipitate and perpetuate these disturbances in the face of possible neuronal injury^29^.

In the studies included in this review of literature, none of them presented the evaluation of daytime symptoms as consequences of insomnia, which can manifest as social and cognitive capacity impairment, besides anxiety^30^. This study shows how feasible and necessary it is to approach HIV-positive patients of advanced age regarding sleep quality and insomnia evaluation. The prevalence of insomnia symptoms in the population studied allows identifying the importance of addressing this disorder in this population. Low et al. (2014)^29^, observed that when studying sleep disorders in HIV patients, it is necessary to consider the CD4 count, viral load, duration, and phase of the disease.

The inclusion criteria adopted in this review minimized the heterogeneity of the studies regarding the samples’ characteristics but caused restrictions on the number of selected studies. Nevertheless, this rigidity allowed the conclusion that these HIV-positive aged patients present a high prevalence of insomnia compared to the general population, suggesting a strong association between the insomnia disorders in the studied patient profile. As limitations of the study, we recognize that in an attempt to select studies oriented toward the diagnosis of insomnia, we excluded the papers that used the Pittsburgh scale, since we did not intend to evaluate the sleep quality but to identify the prevalence of insomnia in older HIV-positive patients. Likewise, we adopted as inclusion criteria studies that report the frequency of insomnia or insomnia symptoms (accepted symptoms: difficulty in starting sleep, difficulty in maintaining sleep, multiple awakenings during sleep, and early awakening). In terms of future perspectives, we suggest new studies that include objective diagnostic criteria for the diagnosis of insomnia in this population.

Thus, the present study concluded that the prevalence of insomnia in HIV-positive elderly patients varied among studies from 12.5% to 76.5%. This significantly higher prevalence, when compared to general population, together with the knowledge on the interaction between inadequate sleep and sleep disorders and immunity dysfunction, suggest that HIV clinical approach should include sleep evaluation in order to improve outcomes and disease prognosis.
